# Difficulties in emotional regulation mediates the impact of burden on quality of life and mental health in a sample of family members of people diagnosed with Borderline Personality Disorder

**DOI:** 10.3389/fpsyg.2023.1270379

**Published:** 2023-11-20

**Authors:** Sara Fonseca-Baeza, Joaquín García-Alandete, José Heliodoro Marco, Sandra Pérez Rodríguez, Rosa M. Baños, Verónica Guillén

**Affiliations:** ^1^Department of Personality, Evaluation and Psychological Treatment, Faculty of Psychology, University of Valencia, Valencia, Spain; ^2^CIBER Fisiopatología Obesidad y Nutrición (CIBEROBN), Madrid, Spain

**Keywords:** Borderline Personality Disorder, family member, burden, emotional regulation, mediation analysis, quality of life, mental health

## Abstract

**Background:**

Although it has been suggested that family members of persons suffering from Borderline Personality Disorder (BPD) endure high levels of burden, however, the process and the impact of this burden in their lives, and specifically the relation between the burden and emotional regulation has not been broadly investigated among this population. The main objective of this study is to examine the impact of burden on quality of life and depression, anxiety and stress, as mediated by difficulties in emotional regulation in family members of persons diagnosed with BPD.

**Method:**

Participants were 167 family members of persons diagnosed with BPD. The Burden Assessment Scale, Difficulties in Emotion Regulation Scale, Multicultural Quality of Life Index, and Depression Anxiety Stress Scale-21 were filled out. Mediation analysis was conducted using the Maximum Likelihood estimator, bootstrap method and listwise deletion for missing data.

**Results:**

Burden showed a significant, negative effect on quality of life and positive on depression, anxiety and stress. Difficulties in emotion regulation significantly mediated these relations. After accounting for the mediating role of difficulties in emotion regulation, burden still had an impact on quality of life, depression, anxiety and stress. Women showed a higher level in both burden and stress than men. The caregivers with secondary and higher studies showed higher levels in burden than those with no studies. Not significant differences in burden, emotion regulation, depression, anxiety and stress were found related to marital status.

**Conclusion:**

Difficulties in emotion regulation mediate the relations between burden and quality of life, depression, anxiety, and stress. Family members could engage in group interventions designed specifically for family members of people with BPD, oriented toward understanding the disorder or learning skills.

## Introduction

It can be challenging to be part of the life of a person with Borderline Personality Disorder (BPD). These persons may experience difficulties in regulating their emotions, affecting their interpersonal relations (Bailey and Grenyer, 2014). These relations are often characterized by constant conflict, physical and verbal aggression, and mood swings. This situation can affect the mental health of family members of persons with BPD. Previous studies have observed that these family members suffer depression, anxiety, and chronic stress (Bennett et al., 2019; Carrotte et al., 2019; Seigerman et al., 2020) at a higher frequency than in the general population (Bailey and Grenyer, 2013; Bennett et al., 2019). In addition, they are affected by the impact that the diagnosis of BPD of their loved one has had on their own lives (Bailey and Grenyer, 2014). On a personal level, there is decreased marital satisfaction because the challenge of adaptation and coping with the disorder makes it difficult to maintain a healthy couple relation, placing affective needs in second place (Goodman et al., 2010; Kay et al., 2018). Likewise, family members of a person diagnosed with BPD also experience difficulties in social relations, mainly as a result of the stigma associated with BPD (Goodman et al., 2010; Ekdahl et al., 2011; Kay et al., 2018). On an occupational level, the impact includes a change in lifestyle or professional career in order to handle the constant demands of persons with BPD (Goodman et al., 2010; Greer and Cohen, 2018). In economic terms, the impact derives from the costs of pharmacological and psychological treatment, but also from the assumption of the daily expenses and the possible debts of the person with BPD (Buteau et al., 2008; Bauer et al., 2012; Kay et al., 2018; de Mendieta et al., 2019). Moreover, occasionally some family members may perceive a severe lack of support and specialised training from the mental health system and the medical community (Ekdahl et al., 2011; Acres et al., 2019; de Mendieta et al., 2019), sometimes feeling misunderstood and stigmatised (Buteau et al., 2008; Lawn and McMahon, 2015; Carrotte et al., 2019).

This situation may contribute to high levels of burden felt by family members of persons diagnosed with BPD (e.g., Giffin, 2008; Bailey and Grenyer, 2013). These high levels of burden may be higher than those felt by family members of persons with other serious mental disorders, such as mood disorders, substance abuse, neurotic disorders, or psychotic disorders, according to some studies (Bailey and Grenyer, 2014, 2015), and are also related to the characteristics of the person with BPD, being higher when the person presents more severe symptomatology (Jørgensen et al., 2021), including conduct problems, verbally abusive behavior, or delusional symptomatology, hallucinations, and comorbid disorders such as anorexia, bipolar disorder, ADHD, or anxiety disorders (Goodman et al., 2010). However, the process and impact of burden on their lives is a topic that has been scarcely explored.

Some studies that have explored the emotional regulation process displayed by family members of persons with BPD, having observed that they may present difficulties in regulating their emotions (Bailey and Grenyer, 2014; Grenyer et al., 2018; Jørgensen et al., 2021). Occasionally, some authors suggest that the regulation process of the family members are more similar to that of patients with post-traumatic stress disorder that the process found in the general population (Bailey and Grenyer, 2014). Emotional regulation can be defined as the cognitive processes that influence the type of emotional response, as well as how individuals experience or express these emotions (Gross, 1998). These processes include the initiation, inhibition, or modulation of internal emotional states, emotion-related cognitions, emotion-related physiological processes, and emotion-related behaviors (Siegler et al., 2020). Furthermore, emotional regulation seems to be involved in the onset and persistence of both physical (e.g., Koechlin et al., 2018; Kupper and Denollet, 2018) and psychological diseases (e.g., Cavicchioli et al., 2021; Muñoz-Navarro et al., 2021; Tyra et al., 2021), as well as on quality of life (Panayiotou et al., 2021).

The relation between burden and emotional regulation has been studied previously, for example in patients with chronic conditions (Schreiner et al., 2021) or family members of persons with cancer (Palacio et al., 2018; O’Toole et al., 2020).

Nevertheless, as far as we know, there are no studies that explore in depth the impact of the burden on the quality of life and mental health of family members of persons with BPD. Only one study has been undertaken that explores the association between burden and emotional regulation, concluding that burden levels are positively correlated with difficulties in emotional regulation (Bailey and Grenyer, 2014).

### The present study

The objective of the present study was to examine the impact of burden on quality of life and depression, anxiety and stress, as mediated by difficulties in emotional regulation in family members of persons diagnosed with BPD. It was hypothesized that burden will negatively predict quality of life and positively predict depression, anxiety and stress. Additionally, it was hypothesized that difficulties in emotional regulation will mediate these relations.

## Method

### Participants

This study used a non-probabilistic convenience sample of 167 parents (mothers: *n* = 120, 71.86%; fathers: *n* = 47, 28.14%) aged between 41 and 75 years old (*M* = 56.54, *SD* = 7.71) of 142 persons diagnosed with BPD. Regarding marital status, 65.87% were married (*n* = 110), 1.20% unmarried but had a partner (*n* = 2), 20.96% divorced (*n* = 35), 7.19% single (*n* = 12), 2.99% widowed (*n* = 5), and 1.80% were missing (*n* = 3). Regarding the educational level, 46.71% had higher education (*n* = 78), 27.54% secondary education (*n* = 46), 10.18% primary education (*n* = 17), 9.58% had no studies (*n* = 16), and 5.99% were missing (*n* = 10 cases).

Regarding the clinical situation of the people diagnosed with BPD, most of them were female (81.7%, *n* = 116) aged between 13 and 64 years old (*M* = 24.7, *SD* = 9.70), 48.4% had a comorbid diagnosis, 89.3% were in psychological treatment, and 88.5% were in pharmacological treatment. The 62.96% of them have had at least one suicidal attempt.

The sample was collected over 4 years (2018–2022) from three Specialized Units for Personality Disorders, from three Associations of Relatives of people with BPD, and from the National Education Alliance for BPD in Spain. The following inclusion criteria were considered for participating in this study: (a) being a parent of a person with a diagnosis of BPD according to the fifth edition of the Diagnostic and Statistical Manual of Mental Disorders (DSM-5) (American Psychiatric Association, 2013), and (b) agreeing and signing an informed consent regarding voluntary participation in the study with no financial incentive. The exclusion criterion was the presence of a serious mental disorder in the family member that required specific specialized care, such a psychosis, schizophrenia, bipolar disorder or substance abuse.

### Instruments

*Burden Assessment Scale* (BAS; Reinhard et al., 1994). We used the García-Alandete et al.’s (2023) Spanish adaptation, a 19-item scale that assesses burden in the process of caregiving in the past 6 months. Burden includes emotions, attitudes, and concerns associated with the caregiver role, as well as reduced personal time or financial problems. Responses are coded on a four-point Likert scale (1 = Not at all; 4 = A lot). In Reinhard et al.’s (1994) study, the BAS showed a good internal consistency: *α* = 0.91 and *α* = 0.89 in two different samples. Higher scores indicate a higher level of burden. Internal consistency of the BAS in this study was *ω* = 0.92 (95% CI [0.91, 0.94]).

*Difficulties in Emotion Regulation Scale* (DERS; Gratz and Roemer, 2004). We used the Spanish adaptation by Hervás and Jódar (2008), a 36-item self-report that assesses different aspects associated with several difficulties in the process of emotional regulation, such as lack of emotional control, lack of emotional attention, life interference, emotional confusion, and emotional rejection. Responses are coded on a five-point Likert scale (1 = Almost never; 5 = Almost always). Higher scores indicate a higher difficulty in the emotional regulation process. In Gratz and Roemer’s (2004) study, the DERS showed a good internal consistency, *α* = 0.93. Internal consistency of the DERS in this study was *ω* = 0.93, 95% CI [0.91, 0.94].

*Multicultural Quality of Life Index* (MQLI; Mezzich et al., 2011). We used the Marco et al.’s (2022) Spanish adaptation, a 10-item scale that assesses physical and emotional well-being, self-care, occupational and interpersonal functioning, socio-emotional and community support, personal and spiritual fulfillment, and an overall perception of quality of life. Responses are coded on a ten-point Likert scale (1 = Bad; 10 = Excellent). Higher scores indicate a higher quality of life. In Mezzich et al.’s (2011) study, the MQLI showed a Cronbach’s α of 0.92. Internal consistency of the MQLI in this study was *ω* = 0.92, 95% CI [0.91, 0.94].

*Depression Anxiety Stress Scale-21* (DASS-21; Lovibond and Lovibond, 1995). It assesses self-perceived physical and subjective symptoms of depression, anxiety, and stress, and an overall perception of these symptoms. Responses are coded on a four-point Likert scale (0 = It did not happen to me; 3 = It happened to me most of the time). Higher scores indicate a higher level of depression, anxiety or stress. Lovibond and Lovibond (1995) found the following Cronbach’s alpha for each scale of the DASS-21: Depression, *α* = 0.91, Anxiety, *ω* = 0.84, and Stress, *α* = 0.90. In this study, internal consistency of the DASS-21 in this study was *ω* = 0.93, 95% CI [0.91, 0.94] for depression, *ω* = 0.91, 95% CI [0.89, 0.93] for anxiety, and *ω* = 0.92, 95% CI [0.90, 0.94] for stress.

### Statistical analysis

First, descriptive statistics and correlations of the scales used in the present study were analyzed. Second, the differences in burden, emotion regulation, quality of life, depression, anxiety, and stress related to gender (*t* test), marital status (ANOVA) and educational level (ANOVA) were analyzed. Third, a mediation analysis using Structural Equation Modeling was conducted to assess if difficulties in emotional regulation mediated the relation between burden and quality of life, depression, anxiety, and stress. Maximum Likelihood estimator, bootstrap method, and listwise deletion for missing data were applied to test the mediational model. The JASP (JASP Team, 2022) software was used for all these analyses.

### Procedure

This study is part of a more extensive research project to implement a skills training program for family members of people with BPD. For this purpose, approval was obtained from the ethics committee of the University of Valencia (INV_ETICA_1955599).

Participants were recruited from three Specialized Units for Personality Disorders, from three Associations of Relatives of people with BPD, and from the National Education Alliance for BPD in Spain. Once the family members were informed about the skills training program and the conditions of the study, those interested signed the informed consent form, and 10 clinical psychologists with experience ranging from 1 to more than 20 years carried out a clinical interview to verify that they met the inclusion and exclusion criteria. The first interviews conducted by the less experienced clinicians were supervised by the more experienced clinicians in order to determine the correct assessment. Finally, they completed the assessment protocol containing the BAS, DERS, MQLI and DASS-21 scales.

During the development of the skills training program, the family members received a specific module on BPD education, where they were introduced to the knowledge of the disorder, the different treatment devices, and the psychological treatments that had empirically demonstrated their efficacy in the treatment of BPD, highlighting that the most empirically supported treatment to date with more than 40 randomized controlled studies is Dialectical Behavior Therapy (Linehan, 1993). Mentalization-Based Therapy (Bateman and Fonagy, 2004), Transference-Focused Psychotherapy (Kernberg et al., 2008) and Schema-Focused Therapy (Young et al., 2003) are also indicated as psychoanalytic therapies that have demonstrated their efficacy (Fassbinder et al., 2016). This information is especially relevant for family members of a person with BPD who is not currently receiving treatment or who is dissatisfied with their usual treatment.

## Results

### Descriptive statistics and correlations

Table 1 shows the descriptive statistics and the correlations of the scales used in the present study. As expected, taking into account what these scales assess, (1) burden was significantly, positively correlated with difficulties in emotion regulation, depression, anxiety and stress, and negatively with quality of life, (2) difficulties in emotion regulation correlated negatively with quality of life, and positively with depression, anxiety and stress (3) quality of life correlated negatively with depression, anxiety and stress, and (4) depression, anxiety and stress correlated positively. Statistically significant values were found in the theoretically expected direction.

**Table 1:** Descriptive statistics and correlations of the scales used in this study.

Scale	*N* (missing)	*M*	*SD*	Pearson’s *r*					
				1	2	3	4	5	6
1. BAS	161 (6)	47.76	13.47	–					
2. DERS	165 (2)	58.44	20.15	0.37***	–				
3. MQLI	164 (3)	62.61	16.63	−0.42***	−0.62***	–			
4. DASS-21-D	166 (1)	0.87	0.82	0.46***	0.63***	−0.61***	–		
5. DASS-21-A	166 (1)	0.62	0.72	0.43***	0.57***	−0.50***	0.72***	–	
6. DASS-21-S	166 (1)	1.08	0.76	0.48***	0.61***	−0.54***	0.81***	0.81***	–

1 = BAS; 2 = DERS; 3 = MQLI; 4 = DASS-21 (Depression subscale); 5 = DASS-21 (Anxiety subscale); 6 = DASS-21 (Stress subscale). BAS, Burden assessment scale; DERS, Difficulties in emotion regulation scale; MQLI, Multicultural Quality of Life Index; DASS-21-D, Depression Anxiety Stress Scale-21 (Depression subscale); DASS-21-A, Depression Anxiety Stress Scale-21 (Anxiety subscale); DASS-21-S, Depression Anxiety Stress Scale-21 (Stress subscale).

***p < 0.001.

### Mediation analysis

Burden was the independent variable, difficulties in emotion regulation was the mediating variable, and quality of life, depression, anxiety and stress were the dependent variables. Regarding the total effects, burden showed a significant, negative effect on quality of life and positive on depression, anxiety and stress (Table 2).

**Table 2:** Total effects.

					95% Confidence Interval	
	Estimate	Std. Error	*z*-value	*p*	Lower	Upper
BAS → MQLI	−0.42	0.07	−5.80	0.000	−0.55	−0.28
BAS → DASS-21-D	0.46	0.07	6.50	0.000	0.31	0.61
BAS → DASS-21-A	0.40	0.07	5.40	0.000	0.26	0.56
BAS → DASS-21-S	0.47	0.07	6.59	0.000	0.34	0.61

Delta method standard errors, bias-corrected percentile bootstrap confidence intervals, ML estimator. BAS, Burden assessment scale; MQLI, Multicultural Quality of Life Index; DASS-21-D, Depression Anxiety Stress Scale-21 (Depression subscale); DASS-21-A, Depression Anxiety Stress Scale-21 (Anxiety subscale); DASS-21-S, Depression Anxiety Stress Scale-21 (Stress subscale).

Analyzing the indirect effects, results revealed that difficulties in emotion regulation significantly, partially mediated the relations between burden and quality of life, depression, anxiety and stress (Table 3).

**Table 3:** Indirect effects.

					95% Confidence interval	
	Estimate	Std. Error	*z*-value	*p*	Lower	Upper
BAS → DERS → MQLI	−0.19	0.05	−4.24	0.000	−0.29	−0.11
BAS → DERS → DASS-21-D	0.20	0.05	4.32	0.000	0.12	0.29
BAS → DERS → DASS-21-A	0.17	0.04	3.94	0.000	0.09	0.26
BAS → DERS → DASS-21-S	0.19	0.04	4.23	0.000	0.11	0.28

Delta method standard errors, bias-corrected percentile bootstrap confidence intervals, ML estimator. BAS, Burden assessment scale; DERS, Difficulties in emotion regulation scale; MQLI, Multicultural Quality of Life Index; DASS-21-D, Depression Anxiety Stress Scale-21 (Depression subscale); DASS-21-A, Depression Anxiety Stress Scale-21 (Anxiety subscale); DASS-21-S, Depression Anxiety Stress Scale-21 (Stress subscale).

The results also suggested that even after accounting for the mediating role of difficulties in emotion regulation, burden still had an impact on quality of life, depression, anxiety and stress (Table 4).

**Table 4:** Direct effects.

					95% Confidence interval	
	Estimate	Std. Error	*z*-value	*p*	Lower	Upper
BAS → MQLI	−0.23	0.07	−3.40	0.000	−0.35	−0.10
BAS → DASS-21-D	0.26	0.06	4.10	0.000	0.14	0.39
BAS → DASS-21-A	0.23	0.07	3.23	0.000	0.11	0.38
BAS → DASS-21-S	0.28	0.07	4.27	0.000	0.15	0.41

Delta method standard errors, bias-corrected percentile bootstrap confidence intervals, ML estimator. BAS, Burden assessment scale; MQLI, Multicultural Quality of Life Index; DASS-21-D, Depression Anxiety Stress Scale-21 (Depression subscale); DASS-21-A, Depression Anxiety Stress Scale-21 (Anxiety subscale); DASS-21-S, Depression Anxiety Stress Scale-21 (Stress subscale).

**Figure 1:**
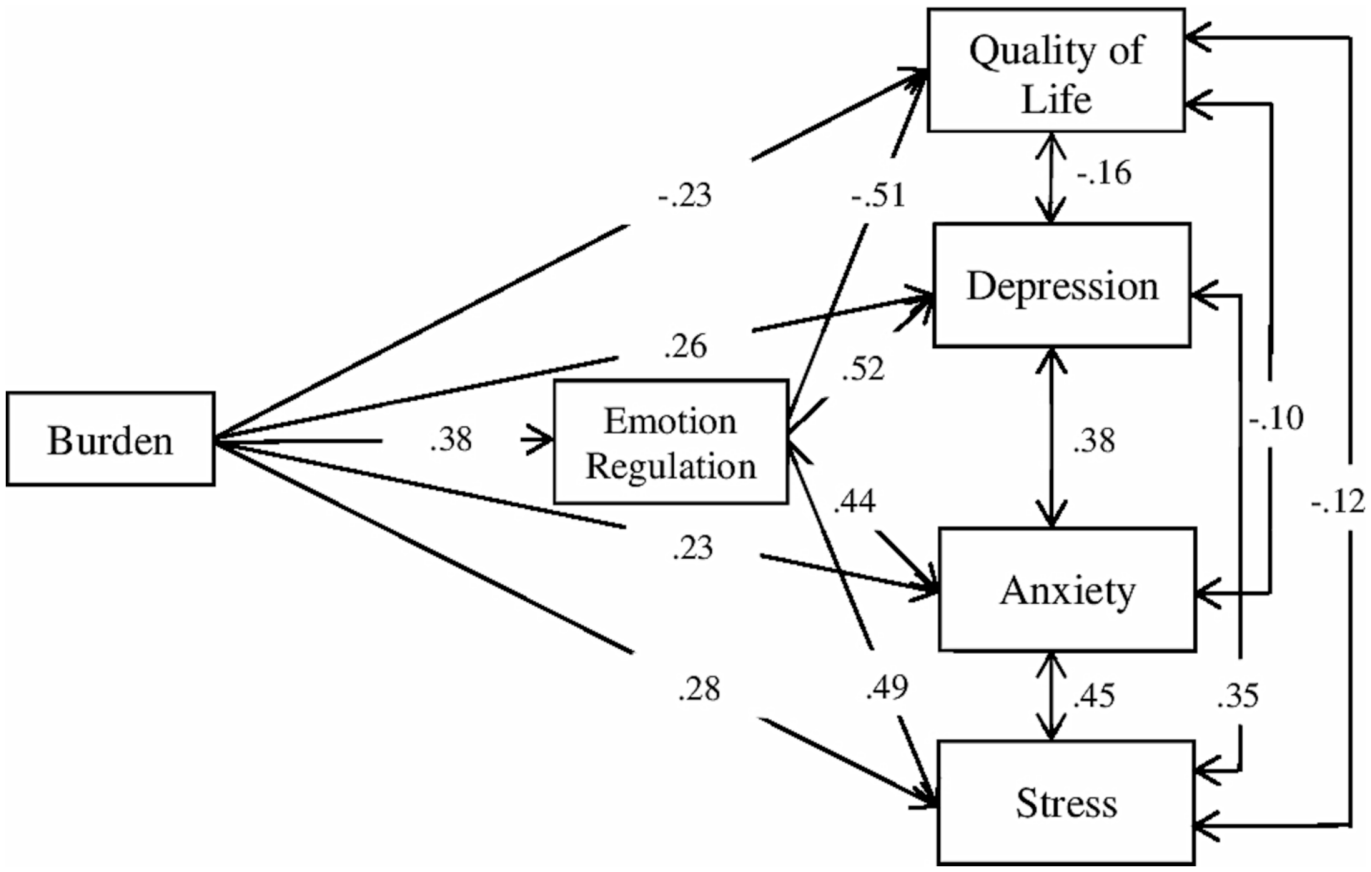
Path plot of the model.

In the tested model (Figure 1), burden explained (*R*^2^) 14% of difficulties in emotion regulation (f2 = 0.16), 40% of quality of life (f2 = 0.67), 45% of depression (f2 = 0.82), 32% of anxiety (f2 = 0.47), and 43% of stress (f2 = 0.75) (according to Cohen’s (2013) guidelines, f2 ≥ 0.02, f2 ≥ 0.15, and f2 ≥ 0.35 represent small, medium, and large effect sizes, respectively).

### Differences related to gender, marital status and educational level in burden, difficulties in emotion regulation, depression, anxiety, and stress

#### Gender related differences

Women showed higher means than men in burden (women: *M* = 49.25, *SD* = 12.76; men: *M* = 44.20, *SD* = 13.75), depression (women: *M* = 0.92, *SD* = 0.89; men: *M* = 0.65, *SD* = 0.86), anxiety (women: *M* = 0.59, *SD* = 0.82; men: *M* = 0.39, *SD* = 0.70) and stress (women: *M* = 1.17, *SD* = 0.84; men: *M* = 0.80, *SD* = 0.79). Men showed higher means than women in difficulties in emotional regulation (women: *M* = 58.25, *SD* = 19.08; men: *M* = 58.88, *SD* = 22.35), and quality of life (women: *M* = 61.90, S*D* = 16.29; men: *M* = 64.35, *SD* = 16.97). Differences were significant for burden [*t*(165) = −2.27, *p* = 0.02, *d* = 0.39] and stress [*t*(165) = −2.661, *p* = 0.01, *d* = 0.45].

#### Marital status related differences

The differences between groups were not significant: burden [*F*(4, 159) = 2.373, *p* = 0.06], difficulties in emotional regulation [*F*(4, 159) = 1.693, *p* = 0.154], quality of life [*F*(4, 159) = 0.820, *p* = 0.514], depression [*F*(4, 159) = 0.672, *p* = 0.612], anxiety [*F*(4, 159) = 0.403, *p* = 0.806], and stress [*F*(4, 159) = 1.719, *p* = 0.148].

#### Educational level related differences

Differences were significant for burden [*F*(3, 153) = 5.262, *p* = 0.002] between those with secondary level studies (*M* = 51.09, *SD* = 13.45) and those with no studies (*M* = 37.06, *SD* = 13.84) (*p* = 0.001, *d* = 0.68), and between those with higher studies (*M* = 49.06, *SD* = 12.05) and those with no studies (*p* = 0.004, *d* = 0.64).

## Discussion

The objective of the present study was to analyze the relations between burden, quality of life, depression, anxiety, and stress in a sample of family members of people diagnosed with BPD and the potential mediating role of difficulties in emotion regulation on this relation. We hypothesized that burden would negatively predict quality of life and positively predict depression, anxiety, and stress. Additionally, it was hypothesized that difficulties in emotion regulation would mediate these relations. The results support these hypotheses.

Results showed that burden was significantly, negatively associated with quality of life, similarly to other studies with relatives of persons with BPD (García-Alandete et al., 2023) or with other pathologies, such as schizophrenia (Ribé et al., 2018; Tristiana et al., 2019), dementia (Schumann et al., 2019; Tulek et al., 2020) or bipolar disorder (Kumar et al., 2019; Ghosh et al., 2020). Furthermore, burden was significantly, positively associated with difficulties in emotional regulation and levels of anxiety, depression, and stress. These results are in consonance with those found in previous literature, both in family members of persons with personality disorders (Bailey and Grenyer, 2014), severe mental disorder (Sun et al., 2019), Alzheimer’s disease (Vespa et al., 2021) or other degenerative processes characteristic of older people (del-Pino-Casado et al., 2021). It is possible that family members who experience a higher burden due to their continued dedication to the care of the person with BPD may give up social and leisure activities that they previously enjoyed, and they may tend to isolate themselves due to the constant demands of caring for the person with BPD (e.g., Greer and Cohen, 2018). This would affect their interpersonal relations, losing social support (e.g., Kay et al., 2018), which has previously been linked in the literature to decreased quality of life (Zhang et al., 2021; Freak-Poli et al., 2022) and impaired mental health (Bjørlykhaug et al., 2021; Schiller et al., 2021). Moreover, the challenges they face not only in the social sphere, but also in the family (e.g., Kay et al., 2018), professional (e.g., Greer and Cohen, 2018) or economic sphere (e.g., de Mendieta et al., 2019), could explain the high presence of stress in these family members. Finally, perhaps this experience of constant burden decreases their ability to cope with the maladaptive behaviors of the person with BPD, increasing their vulnerability to developing secondary traumas associated with the caregiving relation (Bailey and Grenyer, 2014).

Results also showed that difficulties in emotional regulation were significantly, negatively associated with quality of life, and significantly, positively associated with levels of anxiety, depression, and stress. These findings support those obtained by Bailey and Grenyer (2014) in family members of persons with diverse personality disorders, concluding that those with higher difficulties in emotional regulation were also those who presented greater mental health problems, as well as higher levels of overload and grief.

Results indicated that quality of life had a significant, negative relation with levels of depression, anxiety, and stress. This result is consistent with previous findings (e.g., Gan and Yuen Ling, 2019; Babapour et al., 2022). One explicative hypothesis may be that the manifestation of symptoms characteristic of these psychopathologies is what causes the deterioration in quality of life, for example, the worries that constantly dominate the minds of these family members, or the loss of interest in what they previously found pleasurable may be the cause of their diminished well-being.

Finally, the results provided some evidence that family members of persons diagnosed with BPD who do not present difficulties in emotion regulation were more likely to experience a higher quality of life, as well as lower levels of depression, anxiety and stress. It is due to the mediating effect of emotional regulation between burden levels and the quality of life and mental health of the family member. In this regard, Bailey and Grenyer (2015) concluded that family members with low levels of emotional over-involvement have similar levels of burden to family members of persons with other serious mental disorders, such as mood disorders, substance abuse, neurotic disorders, and psychotic disorders. However, family members with high emotional over-involvement have levels of burden higher than one standard deviation of the levels of burden of family members of persons diagnosed with other pathologies. Perhaps relatives with greater emotion dysregulation have difficulties in maintaining a low emotional involvement in the relation with the person with BPD, and this over-involvement would be promoting a higher perception of burden in the caregiving process.

These results bring about a closer understanding of the effects that the process of caring for people diagnosed with BPD has on their family caregivers, and provide insight on where to intervene. This is also important for the recovery of persons diagnosed with BPD, since the perceived burden, quality of life and mental health of the persons involved in the caregiving process have been related to a deterioration in caregiving performance (e.g., Babapour et al., 2022). Conversely, an adequate emotional involvement in the family and in the affected person has been related to better clinical outcomes, such as less hospitalizations and an improved family environment (Hooley and Hoffman, 1999).

### Differences related to gender, marital status and educational level in burden, difficulties in emotion regulation, depression, anxiety, and stress

Regarding the relationships between burden, difficulties in emotion regulation, depression, anxiety, and stress, and sociodemographic variables, the results obtained in this study showed the following.

#### Gender

Women showed a higher level in both burden and stress than men. This result support the obtained in previous studies (e.g., Papastavrou et al., 2009; Udoh et al., 2021). One hypothesis is that women are more dedicated than men to the care of their relatives, especially when these relatives suffer a mental health. The social roles commonly associated with gender (women are more dedicated to the care of the home and family; men are more oriented to the external and professional sphere) would condition women to be much more aware than men of the attention and care of their relatives that suffer a mental disorder and, consequently, and therefore suffer a higher level of burden and stress. Previous studies report that men have better quality of life than women in the physical domain, overall quality of life, bodily pain, general health, vitality, and mental health (e.g., Lima-Rodríguez et al., 2022).

#### Studies level

The caregivers with secondary and higher studies showed higher levels in burden than those with no studies. This result is contrary to that found in other studies. For example, Lima-Rodríguez et al. (2022) report that several studies found that physical, psychological, social, and environment domains were greater at higher educational levels. One hypothesis is that people with higher levels of education have greater dedication and professional development than those with lower levels of education. It could be that people with higher levels of education experience a greater clash between their professional commitments and aspirations and the demands of caring for a family member with a mental disorder than those with lower levels of education and a lower job profile.

#### Marital status

Regarding the marital status, we found not significant differences between groups in burden, emotion regulation, depression, anxiety and stress. This result is contrary to the results of previous studies that found negative effects of caring for relatives with severe mental disorders. For example, Udoh et al. (2021) found a higher mean burden of care among participants categorized as separated and widowed, compared with the married and participants who were single. In our study, the non-significance of differences may be explained, in part, by the small sample size of single, widowed and partnered but unmarried individuals and, in part, by the fact that divorced individuals may share the care of their BPD family member as well as married individuals. The role of marital status in the impact of caring for a family member with BPD on caregivers should be specifically studied.

### Clinical implications

This study has several clinical implications. Firstly, it may be noted that the relation between the caregiving process and the mental health of family caregivers is more complex than it may seem at first glance, and an exhaustive study of the relations between all the variables involved is necessary.

Secondly, it is shown that reducing the burden associated with caring for a relative diagnosed with BPD, for example by reducing the family caregivers’ working hours or increasing their financial resources, will not have a substantial impact on their symptomatology if emotional dysregulation is not also reduced. This is because emotional dysregulation mediates the impact of burden on symptomatology, making the perception of burden greater or lesser -regardless of its objective value- and having a greater or lesser impact on the family member’s symptomatology. Therefore, reducing load levels without reducing emotional dysregulation will not have the effects on the elimination of symptomatology that might have been expected (e.g., Bailey and Grenyer, 2014).

Thirdly, it raises the possibility of protecting the mental health of family members through interventions that promote emotional regulation, such as Acceptance and Commitment Therapy (Hayes et al., 2009), Dialectical Behavioral Therapy (Linehan, 1993), Mindfulness (Roemer et al., 2015), Unified Protocol (Barlow et al., 2017) or Systems Training for Emotional Predictability and Problem Solving (Blum et al., 2002). This is especially relevant in the case of family members of persons with BPD, where, due to the symptomatology of the disorder, the family member has less control over the things that happen in his or her life. Likewise, family members could engage in group interventions designed specifically for family members of people with BPD, oriented toward understanding the disorder (e.g., Pearce et al., 2017; Grenyer et al., 2018) or learning skills (e.g., Bateman and Fonagy, 2019; Fonseca-Baeza et al., 2021). Of these interventions, Family Connections (Hoffman et al., 2005) has received the most empirical support to date (Guillén et al., 2020; Sutherland et al., 2020).

### Limitations and suggestions for further research

A methodological strength of the present study was to carry out the mediation analysis using SEM and bootstrapping methods. SEM provides a more appropriate inference framework for mediation analyses than standard regression methods (Gunzler et al., 2013). Bootstrapping method has higher statistical power and control on type-I error than classical methods (Bollen and Stine, 1990; Dastgeer et al., 2021). Despite this strength, this study has a number of limitations that should be emphasized. The research design was cross-sectional. It would be interesting to carry out longitudinal studies controlling for changes in some parameters of patients’ status and behavior (e.g., severity of the disorder, suicide attempts, years of evolution, etc.) in order to assess whether changes occur in the quality of life and mental status of family caregivers. Some variables of the family caregivers that could be relevant may not have been taken into account in the analyses, such as their capacity for resilience, hope or acceptance (Wang et al., 2020; Calvete et al., 2021; García-Castro et al., 2021); or regarding the positive consequences associated with the caregiving process itself (Seigerman et al., 2020; García-Castro et al., 2021). In this sense, Fauziana et al. (2018) concluded that positive caregiving characteristics mediates the impact of the burden in the satisfaction with life in family members who cares an older adult.

The sample was composed only of parents of persons with BPD, so the results cannot be generalized to other family (e.g., siblings, aunts, uncles, children, etc.). Even more, the sample only included Spanish participants, who participate in a Mediterranean family culture (e.g., Moreno-Cámara et al., 2019). It could be interesting to include participants from other cultures in which family ties may be not as strong and neither are the obligations of some family members with respect to others, especially with those who require particular attention and care due to a severe mental disorder.

It would be important to delve into the relationship between gender, educational level and professional demand or dedication (and their interrelationships), and the burden of caring for a family member with a mental disorder.

Further progress in understanding the implications for family members of caring for a person with BPD or other personality disorders is essential. It is needed to identify those factors involved in the caregiving process and provide a detailed picture of how and where interventions could be made to reduce the impact on the family member.

## Conclusion

In summary, this study suggests that difficulties in emotion regulation mediate the relations between burden and quality of life, depression, anxiety, and stress in family members of persons diagnosed with BPD. Future research could also analyze the impact of other variables involved. Nevertheless, emotion regulation should be a therapeutic target of intervention programs for family members.

## Data availability statement

The raw data supporting the conclusions of this article will be made available by the authors, without undue reservation.

## Ethics statement

The studies involving humans were approved by Ethics Committee of the University of Valencia. The studies were conducted in accordance with the local legislation and institutional requirements. Written informed consent for participation was not required from the participants or the participants' legal guardians/next of kin in accordance with the national legislation and institutional requirements. Written informed consent was obtained from the individual(s) for the publication of any potentially identifiable images or data included in this article.

## Author contributions

SF-B: Writing – original draft, Writing – review & editing. JG-A: Writing – original draft, Writing – review & editing. JM: Writing – original draft, Writing – review & editing. SP: Writing – original draft, Writing – review & editing. RB: Writing – original draft, Writing – review & editing. VG: Writing – original draft, Writing – review & editing.
